# Supplementary material for: Healthcare waste management practices and associated factors among healthcare workers in Sub-Saharan Africa: A systematic review and meta-analysis
Source: PLoS One. 2025 Oct 13;20(10):e0334290. doi: 10.1371/journal.pone.0334290 (PMC12517534; doi:10.1371/journal.pone.0334290)
Supplement: S2 File — (DOCX) [file pone.0334290.s002.docx]

**Results of JBI Quality Assessment**

| Studies | Clear eligibility criteria | Description of study subject and study setting | Valid and reliable method to measure the exposure | Standard criteria used for measurement of the condition | Identification of confounding factors | Develop of strategies to deal with confounding factors | Valid and reliable method to measured outcomes | Appropriate statistical analysis | Total score out of 8 | Quality of score (100%) |
| --- | --- | --- | --- | --- | --- | --- | --- | --- | --- | --- |
| Assemu et.al (2020) | Yes | Yes | Yes | Yes | Yes | Yes | Yes | Yes | 8 | 100 |
| Alice et.al (2023) | No | No | Yes | Yes | Yes | Yes | Yes | Yes | 6 | 75 |
| Doylo et.al (2019) | No | Yes | Yes | Yes | Yes | Yes | Yes | Yes | 7 | 87.5 |
| Babirye et.al (2020) | No | Yes | Yes | Yes | Yes | Yes | Yes | Yes | 7 | 87.5 |
| Tolesa (2019) | Yes | Yes | Yes | Yes | Yes | Yes | Yes | Yes | 8 | 100 |
| Berhanu et.al (2022) | No | Yes | Yes | Yes | Yes | Yes | Yes | Yes | 7 | 87.5 |
| Wafula et.al (2019) | No | Yes | Yes | Yes | Yes | Yes | Yes | Yes | 7 | 87.5 |
| Mariam et.al (2018) | Yes | Yes | Yes | Yes | Yes | Yes | Yes | Yes | 8 | 100 |
| Gizalew et.al (2021) | Yes | Yes | Yes | Yes | Yes | Yes | Yes | Yes | 8 | 100 |
| Ibrahim et.al (2023) | Yes | Yes | Yes | Yes | Yes | Yes | Yes | Yes | 8 | 100 |
| Tilahun et.al (2023) | No | Yes | Yes | Yes | Yes | Yes | Yes | Yes | 7 | 87.5 |
| Mitiku et.al (2022) | Yes | Yes | Yes | Yes | Yes | Yes | Yes | Yes | 7 | 87.5 |
| Sahiledengle (2019) | Yes | Yes | yes | Yes | Yes | Yes | Yes | Yes | 8 | 100 |
| Ekanem et.al (2021) | Yes | Yes | Yes | Yes | Yes | Yes | Yes | Yes | 8 | 100 |
| Rutayisire et.al (2019) | No | Yes | Yes | Yes | Yes | No | Yes | Yes | 6 | 75 |
| Woromogo et.al (2020) | Yes | No | Yes | Yes | Yes | No | Yes | Yes | 5 | 62.5 |
| Deress et.al (2018) | Yes | yes | yes | yes | yes | Yes | yes | yes | 8 | 100 |
| Olifa et.al (2018) | Yes | Yes | Yes | Yes | Yes | Yes | Yes | Yes | 8 | 100 |
| Wassie et.al (2022) | No | Yes | Yes | Yes | No | No | Yes | Yes | 5 | 62.5 |
| Yakubu et.al (2023) | No | Yes | Yes | Yes | No | No | Yes | Yes | 5 | 62.5 |
| Millanzi et.al (2023) | Yes | Yes | Yes | Yes | No | No | Yes | Yes | 6 | 75 |
| Leonard et.al (2022) | No | Yes | Yes | Yes | No | No | Yes | Yes | 5 | 62.5 |
| Deress et.al (2019) | No | Yes | Yes | Yes | No | No | Yes | Yes | 5 | 62.5 |
| Lemma et.al (2023) | Yes | Yes | Yes | Yes | No | No | No | No | 6 | 75 |
| Salaam et.al (2022) | No | Yes | Yes | Yes | No | No | Yes | Yes | 5 | 62.5 |
| Thankam et.al (2021) | Yes | Yes | Yes | Yes | No | No | Yes | Yes | 6 | 75 |
| Mekassa et.al (2022) | No | Yes | Yes | Yes | Yes | Yes | Yes | Yes | 7 | 87.5 |
| Mogaka et.al (2023) | Yes | Yes | Yes | Yes | No | No | Yes | Yes | 6 | 75 |
| Onuh et.al (2019) | Yes | Yes | Yes | Yes | No | No | Yes | Yes | 6 | 75 |
