# Supplementary material for: Healthcare waste management practices and associated factors among healthcare workers in Sub-Saharan Africa: A systematic review and meta-analysis
Source: PLoS One. 2025 Oct 13;20(10):e0334290. doi: 10.1371/journal.pone.0334290 (PMC12517534; doi:10.1371/journal.pone.0334290)
Supplement: Attachment — Submitted filename: Review report.docx [file pone.0334290.s004.docx]

**Date: 15 September 2024**

**Manuscript Title:** Healthcare waste management practice and associated factors among healthcare workers in Sub-Saharan Africa: a systematic review and meta-analysis

**Journal:** PLOS ONE

**Manuscript ID:** PONE-D-24-32196

**Manuscript type**: Review article

**GENERAL COMMENTS:**

Authors reviewed a topic which is highly relevant for healthcare workers, practitioners, regulators, and policy makers regarding healthcare waste management. The methodology and results are very good. However, the articles included in the review are mainly from East Africa/Ethiopia. Hence, further literature search and analysis is required if the term SSA is included in the title of the manuscript. Therefore, the manuscript can be accepted for publication after major revision.

**SPECIFIC COMMENTS:**

**Introduction:**

1. Authors cited local studies for global figures which is not appropriate. For example, line 61-63: “Annually, 5.2 million people worldwide, including 4 million children, death are reported as a result of improper management [7]. Additionally, more than 2 million healthcare workers are infected by over 30 dangerous blood - borne pathogens [4]. Reference number 4 and 7 are a study from Ethiopia but cited for the global public health burden associated with poor healthcare waste management..
2. Check the validity of the statement from line 64-66: “Exposure to hazardous waste containing mercury and expired drugs can lead to serious health issues like cancer, mutations, teratogenicity, and infections of the eyes, respiratory system, and skin diseases [9]” How infection of the eye and the like from mercury and expired drugs exposure.
3. Line 69-74 discusses improper disposal of healthcare waste such as indiscriminate dumping, burying and burning as well as the associated health and environmental risks. Thes practices are not related to healthcare workers. These issues are applicable for management bodies and medical waste handlers/janitors. It’s better to remove this part.
4. The introduction lacks healthcare workers waste management practice information. Authors reviewed mainly risks associated with poor waste management practices.

**Materials and Methods:**

5. On page 4, Line 106: Delete the subtitle “Study Setting: The study setting is SSA.” As it is already indicated in the manuscript title and in line 92-93 on the same page.
6. Among 18 articles included in the meta-analysis, more than 60% were from Ethiopia. When articles from Ethiopia, Ruanda and Uganda combined, they account for 83% from the total articles in the SSA countries. Is it appropriate to mention SSA in the title? If it appears in the title, more articles are required from other SSA countries. Authors compared their review result with the study conducted in Kenya [“………… the finding of this review was lower than the studies done in Kenya (72.5%) [43]”] but did not include it in the review. Why?

| **Country** | **Number of included articles** | **%** |
| --- | --- | --- |
| Ethiopia | 11 | 61.1 |
| Ruanda | 2 | 11.1 |
| Uganda | 2 | 11.1 |
| **East Africa** | 15 | 83.3 |
| Nigeria | 1 | 5.6 |
| Cameron | 1 | 5.6 |
| South Africa | 1 | 5.6 |
| Total | 18 | 100 |

7. Authors reach a conclusion based on the ‘Good Practice’ report of each included study. However, the operational definition of ‘Good Practice’ might not be similar for all studies. This will have implications for the validity and reliability of the method to measure the outcome variable. This need clarification.

**Discussion:**

8. Page 14: “This review also revealed that the sex of the healthcare workers, the presence of healthcare waste management manuals/ guidelines in their working departments, the knowledge status of the healthcare workers, the presence of training on healthcare waste management practices, and working hours per a day were factors significantly associated with healthcare waste management practices among healthcare workers.” Is it a result or a discussion?
9. In the last paragraph: though the findings are compared with similar study results, justification for favoring or discouraging good practices were not mentioned.
10. No comparison is made with studies from North African countries, Latin America countries and the like.

**Conclusions and Recommendations:**

11. “This review revealed that the pooled estimated good practices of HCWM among healthcare workers in SSA were relatively low.” What does ‘relatively low mean’?
12. “The sex of the healthcare worker, the presence of waste management manuals/guidelines, knowledge of healthcare workers on healthcare waste management, training on waste management, and the working hours per day were factors significantly associated with HCWM practices among healthcare workers.“ Is it being male or female that tends to good practice? Is working less than or more than 8 hours that favors good practice? Show the direction of each factor towards good practice.
