# Supplementary material for: Healthcare waste management practices and associated factors among healthcare workers in Sub-Saharan Africa: A systematic review and meta-analysis
Source: PLoS One. 2025 Oct 13;20(10):e0334290. doi: 10.1371/journal.pone.0334290 (PMC12517534; doi:10.1371/journal.pone.0334290)
Supplement: Attachment — Submitted filename: Rebuttal letterssss.docx [file pone.0334290.s005.docx]

**Rebuttal letter**

Journal Requirements

#1. When submitting your revision, we need you to address these additional requirements.

Please ensure that your manuscript meets PLOS ONE's style requirements, including those for file naming. The PLOS ONE style templates can be found at

https://journals.plos.org/plosone/s/file?id=wjVg/PLOSOne_formatting_sample_main_body.pdf and

**Response: we have incorporated all the requirement as per the standards of the PLOS ONE.**

#2. Please include captions for your Supporting Information files at the end of your manuscript, and update any in-text citations to match accordingly. Please see our Supporting Information guidelines for more information: <http://journals.plos.org/plosone/s/supporting-information>.

**Response: Thank you very much we have modified the revised version of the manuscript based on its requirements.**

**Reviewer 1**

**#1.** Authors cited local studies for global figures which is not appropriate. For example, line 61-63: “Annually, 5.2 million people worldwide, including 4 million children, death are reported as a result of improper management [7]. Additionally, more than 2 million healthcare workers are infected by over 30 dangerous blood - borne pathogens [4]. Reference number 4 and 7 are a study from Ethiopia but cited for the global public health burden associated with poor healthcare waste management.

**Response: Sorry for the confusion we have created. Hence, we have modified the references accordingly in the revised version of the manuscript.**

**#2.** Check the validity of the statement from line 64-66: “Exposure to hazardous waste containing mercury and expired drugs can lead to serious health issues like cancer, mutations, teratogenicity, and infections of the eyes, respiratory system, and skin diseases [9]” How infection of the eye and the like from mercury and expired drugs exposure.

**Response:** **Sorry for the confusion we have created. We have rechecked the validity of the statement from the references I got it and it is a valid statement. The statement focus on the effects of exposing on hazardous waste containing mercury.**

#3. Line 69-74 discusses improper disposal of healthcare waste such as indiscriminate dumping, burying and burning as well as the associated health and environmental risks. These practices are not related to healthcare workers. These issues are applicable for management bodies and medical waste handlers/janitors. It’s better to remove this part.

**Response: Thank you very much for your critical insights to improve the quality of the manuscript. Hence we have amended the revised manuscript based on your suggestion.**

#4. The introduction lacks healthcare workers waste management practice information. Authors reviewed mainly risks associated with poor waste management practices.

**Response: thank you very much for your concern. Hence, we have used additional references which add pertinent information on healthcare waste management practices.**

#5. On page 4, Line 106: Delete the subtitle “Study Setting: The study setting is SSA.” As it is already indicated in the manuscript title and in line 92-93 on the same page.

**Response: Sorry for the confusion we have created. Hence we have removed the subtitle study setting in the revised manuscript.**

#6. Among 18 articles included in the meta-analysis, more than 60% were from Ethiopia. When articles from Ethiopia, Ruanda and Uganda combined, they account for 83% from the total articles in the SSA countries. Is it appropriate to mention SSA in the title? If it appears in the title, more articles are required from other SSA countries. Authors compared their review result with the study conducted in Kenya [“………… the finding of this review was lower than the studies done in Kenya (72.5%) [43]”] but did not include it in the review. Why?

**Response: Sorry for the confusion we have created. We have tried our best to find out more literatures done in various SSA. Regarding the inclusion of SSA in the title, one of the advantages of using systematic review and meta-analysis is the use of pre-determined inclusion criteria to reduce the burden of bias. We have tried to find out more literatures in different countries across the continent to reduce your concern as much as possible. Various researches were conducted on healthcare waste management practices among healthcare workers, but they failed to qualify the inclusion criteria. The other critical concern is the use of literatures done in Kenya to compare the finding of this review but should be part of the review, we have amended it in the revised manuscript hence Kenya is already part of SSA.**

#7. Authors reach a conclusion based on the ‘Good Practice’ report of each included study. However, the operational definition of ‘Good Practice’ might not be similar for all studies. This will have implications for the validity and reliability of the method to measure the outcome variable. This need clarification.

**Response: Thank you very much for your critical insight. To overcome the problem you have mentioned we have used literatures which operationalize as having of good healthcare waste management practices whey the perform more than 50% of the recommended healthcare waste management practices when implementing greater than or equal to the mean score of the practice measures.**

#8. Page 14: “This review also revealed that the sex of the healthcare workers, the presence of healthcare waste management manuals/ guidelines in their working departments, the knowledge status of the healthcare workers, the presence of training on healthcare waste management practices, and working hours per a day were factors significantly associated with healthcare waste management practices among healthcare workers.” Is it a result or a discussion?

**Response: thank you very much for your concern. It is obviously clear that the above statement is more of a result section. However, in discussion the there is a trend to present the overall finding in a precise manner as introduction part of the discussion. If you believe that this is not important, we can omit it from the place**

#9. In the last paragraph: though the findings are compared with similar study results, justification for favoring or discouraging good practices were not mentioned.

**Response: Sorry for the problems we have created. Hence we have included justification for favoring or discouraging good practices in the revised version of the manuscript.**

#10. No comparison is made with studies from North African countries, Latin America countries and the like.

**Response: thank you very much for your critical insights, hence we have tried our best to include literatures done in North African and Latin America. See the revised version of the manuscript.**

#11. “This review revealed that the pooled estimated good practices of HCWM among healthcare workers in SSA were relatively low.” What does ‘relatively low mean’?

**Response: Sorry for the confusion we have created. Hence we have modified the way expression in the revised version to overcome this confusion. See the revised version of the manuscript.**

#12. “The sex of the healthcare worker, the presence of waste management manuals/guidelines, knowledge of healthcare workers on healthcare waste management, training on waste management, and the working hours per day were factors significantly associated with HCWM practices among healthcare workers.“ Is it being male or female that tends to good practice? Is working less than or more than 8 hours that favors good practice? Show the direction of each factor towards good practice.

**Response: Thank you very much for your concern. All the reference variables are presented in brackets. The outcome of the study was expresses in terms of good practices. For example in this study female is presented as a references and the odd is greater than one which implies that being male is odd ratios times more likely to implement a good healthcare waste management practices than females. Similar principles will be applied for all other variables which have been declared as factors significantly associated with the outcome variables of the study.**

**Reviewer 2**

**#1.** The pooled estimate of HCWM practices (50.42%) is based on studies with very high heterogeneity (I² = 97.7%). While subgroup analyses attempt to explore sources of heterogeneity, significant variability remains, limiting the generalizability of the results. Pooling data across diverse countries with varying healthcare systems and policies may mask important contextual factors influencing HCWM practices

**Response: thank you very much for your critical concern. Hence to find out the sources of heterogeneity we have performed meta-regression. The study setting is which is expected to share various aspects in terms of healthcare waste management practices. Therefore, for overcome such types of diversification, it is advisable to carry out Meta regression to determine the sources of heterogeneity. See the revised version of the manuscript.**

#3. The exclusion of qualitative studies limits a deeper understanding of barriers and facilitators to HCWM practices, which is critical for policy and practice recommendations.

**Response: Thank you very much for your critical concern. We have already excluded studies done o qualitative studies. This is because they have their own methods of systematic review and meta-analysis. Therefore, it is difficult to manage both qualitative and quantitative studies simultaneously in a systematic review and meta-analysis. So we believe that systematic review for qualitative review should be done independently.**

#4. Address the high heterogeneity more thoroughly by exploring meta-regression or other advanced statistical methods. Consider discussing the potential limitations of pooling data across diverse contexts in more depth. Appropriate statistical techniques were used, including random-effects modeling, Egger’s regression test for publication bias, and sensitivity analyses. Subgroup analyses by country, healthcare facility ownership, and pre- versus post-COVID-19 pandemic periods add depth to the findings. Despite subgroup analyses, high heterogeneity persists, which undermines the reliability of the pooled estimates. The justification for choosing the random-effects model is not explicitly stated, and alternative approaches (e.g., mixed-effects models) are not considered. The authors could have performed a meta-regression to explore potential moderators contributing to the observed heterogeneity. Provide a clearer rationale for model selection and explore advanced methods to account for heterogeneity. This would enhance the robustness of the findings.

**Response: Thank you very much for your critical insight. Hence, we have conducted a Meta regression for find out the sources of heterogeneity. Additionally, we have included justification for selection of appropriate models in conducting systematic review and meta-analysis.**

#5. The authors have stated that all relevant data are fully available within the manuscript and supporting information. References to included studies and the search strategy are provided, ensuring transparency and compliance with PLOS ONE’s data policy. The manuscript communicates the main findings and methodology effectively. There are several grammatical errors, typographical mistakes, and instances of unclear phrasing that impact the readability and professionalism of the manuscript. Examples include: Overly complex sentences, such as “The pooled estimate of good HCWM practices among study participants was found to be 50.42%...” These could be simplified for clarity.

**Response: sorry for the confusion we have created. We have amended all the issues you have raised. See the revised version of the manuscript.**

#7. Repetitive phrases, such as “good healthcare waste management practices,” could be varied to improve flow.

**Response: thank you very much for your suggestion. Hence, we have tried to amend the problem in revised version of the manuscript.**

#8. Ambiguities in phrasing, such as “may cause the missing of some important studies,” should be revised to “may result in the omission of important studies

**Response: sorry for the confusion we have created. Hence we have modified such types of unclear ideas throughout the manuscript. See the revised manuscript**

#9. The manuscript requires editing for grammar, syntax, and readability to meet the standard of clear and unambiguous English required by PLOS ONE. A professional language editing service may be beneficial.

**Response: Thank you very much for your concern. Hence, we have amended the manuscript considering the PLOS ONE journal requirements and you suggestions. See the revised version of the manuscript.**

#10. The manuscript outlines some strengths, such as the use of PRISMA guidelines, comprehensive literature search, and robust inclusion criteria. However: The exclusion of non-English studies may introduce bias and limit the generalizability of findings, particularly in multilingual regions. The reliance on quantitative studies excludes valuable qualitative insights that could enrich understanding.

**Response: thank you very much for your concern. We have excluded studies done in languages other than English language as a pre-determined criteria which is one of the strength of using a systematic review and meta-analysis. It is obviously clear than that we may miss pertinent findings done in other language. Because we do not have experts with other languages in understand their findings. Therefore, we have presented it as one of the limitation of this systematic review and meta-analysis. See the revised version of the manuscript.**

#11. Expand the discussion of the study’s limitations, particularly regarding heterogeneity, language restrictions, and the exclusion of qualitative studies. This would provide a more balanced view of the study’s findings. No concerns regarding research ethics or dual publication were identified.

**Response: thank you very much for your concern. Hence, we have included your suggestion in the revised version of the manuscript.**

#12. The forest plots and PRISMA diagram are informative but could benefit from improved labeling to enhance clarity for readers.

**Response: Thank you very much for your concern. Hence we have tried to present the figures based on the requirements of the journal using a PACE application for editing of graphs in PLOS ONE.**

#13. While the authors provide recommendations for improving HCWM practices, these could be strengthened by incorporating actionable steps tailored to specific contexts (e.g., low-resource settings)

**Response: thank you very much for your concern. Hence, we have modified the recommendation as per your suggestion. See the revised version of the manuscript.**

**Reviewer 3**

**#1.** Enhance the introduction by clearly articulating the novel contributions of the study, such as new insights into the factors influencing healthcare waste management practices in this specific regional context.

**Response: thank you very much for your critical concern. Hence, we have modified the introduction as per your recommendation. See the revised version of the manuscript.**

**#2.** I recommend using abbreviations sparingly, only for frequently repeated terms, or providing an abbreviation guide with the full meanings.

**Response: sorry for the confusion we have created. Hence we have tried to use abbreviations and acronyms to lowers level. See the revised version of the manuscript.**

#3. The reported high heterogeneity (I² = 97.7%) suggests substantial variability in the study outcomes, which could impact the generalizability of the findings. A more detailed exploration of the sources of this heterogeneity could strengthen the conclusions.

**Response: thank you very much for your concern. To overcome the sources of heterogeneity, we have performed meta-regression to enhance the generalizability of the findings. See the revised version of the manuscript.**

#4. Some grammatical phrases need revision to enhance clarity and readability, e.g., "However, during by the two independent reviewers, the issues were resolved through an evidence-based discussion

**Response: thank you very much for your concern. We have incorporated your comments in the revised version of the manuscript. See the revised version of the manuscript.**
