# Supplementary material for: Healthcare waste management practices and associated factors among healthcare workers in Sub-Saharan Africa: A systematic review and meta-analysis
Source: PLoS One. 2025 Oct 13;20(10):e0334290. doi: 10.1371/journal.pone.0334290 (PMC12517534; doi:10.1371/journal.pone.0334290)
Supplement: Attachment — Submitted filename: Response to reviewers.docx [file pone.0334290.s006.docx]

**Rebuttal letter two**

**Response to the editor and reviewers.**

**Reviewer 1:** has no concerns**.**

**Response to Reviewer 2**

**#1.** The meta-analysis continues to exhibit very high heterogeneity (I² = 97.7%), which is only partially addressed through subgroup analyses and meta-regression. Although the authors explored variability by country, facility ownership, and the COVID-19 period, residual heterogeneity remains substantial. This weakens the strength of the pooled estimate and limits the generalizability of the findings. Moreover, the inclusion of studies from diverse healthcare systems with varying definitions of “good HCWM practices” introduces further contextual variability that is not fully accounted for in the interpretation. The decision to exclude non-English and qualitative studies—while methodologically valid—reduces the comprehensiveness of the evidence base. Policy recommendations, though revised, are still somewhat generic and not consistently anchored in the patterns identified through analysis.

The authors should elaborate on the meta-regression findings by reporting key statistics such as p-values, regression coefficients, and the proportion of variance explained (R²). Additionally, they should explicitly discuss how differences in healthcare systems and definitions of “good HCWM practices” across countries may limit the generalizability of the pooled estimate.

**Response: Thank you sincerely for your valuable and constructive feedback aimed at improving the quality of our manuscript. As highlighted in the results section, the heterogeneity among the included studies was notably high, with an I² value approaching 97%, indicating substantial variability. To explore potential sources of this heterogeneity, we conducted subgroup analyses; however, none of the examined variables accounted for the observed variation. Consequently, we proceeded with a meta-regression analysis to further investigate the origins of heterogeneity. As presented in the meta-regression table, all variables exhibited confidence intervals that spanned both negative and positive values, suggesting no significant contribution to heterogeneity. This interpretation is further supported by the p-values, all of which exceeded the conventional threshold of statistical significance (p > 0.05), reinforcing the conclusion that the variables assessed were not responsible for the heterogeneity. Regarding outcome measurement, although the included studies employed various assessment tools, the majority defined "good practice" as achieving a score of 50% or higher on the recommended preventive measures. Accordingly, this has been acknowledged as one of the limitations of the present systematic review and meta-analysis**

**#2.** The authors have strengthened the statistical methods in the revised version by applying random-effects modelling, performing subgroup analyses and meta-regression, and assessing publication bias and sensitivity. However, the rationale for selecting specific models, such as the random-effects approach over mixed-effects or Bayesian models, could be more thoroughly articulated. Importantly, the results of the meta-regression are not adequately detailed—regression coefficients, p-values, and explained variance (e.g., R²) are not reported, limiting the reader’s ability to assess its effectiveness. Confidence intervals for subgroup estimates and heterogeneity statistics within subgroups should also be reported and discussed more explicitly. Finally, assumptions underlying the statistical models are not addressed.

While random-effects models are appropriate, the rationale for their selection over alternative models (e.g., mixed-effects or Bayesian approaches) should be explained more clearly. The manuscript should also provide more detailed reporting of subgroup analyses, including subgroup-level heterogeneity (I²) and confidence intervals for each pooled estimate.

**Response: The fixed effect model and random effects model are the two most commonly used approaches in systematic reviews and meta-analyses. Since this systematic review and meta-analysis focus on Sub-Saharan African countries, it is anticipated that there will be variability in the factors contributing to the outcomes. The fixed effect model is appropriate when the study populations are relatively homogeneous regarding these contributing factors. However, our research encompasses a vast geographic region that is likely to exhibit substantial heterogeneity. For this reason, the random effects model is recommended to assess the degree of heterogeneity across studies. When heterogeneity is minimal, typically less than 25%, the fixed effect model is preferable. Conversely, if heterogeneity is substantial, a random effects model is more suitable. In our analysis, the level of heterogeneity is very high, close to 97%, which justifies the use of the random effects model in our study.**

**#3.** The current Data Availability Statement does not fully comply with PLOS ONE’s requirements. While the authors state that all relevant data are within the manuscript and Supporting Information, no raw data (e.g., extracted prevalence rates, sample sizes, standard errors) are provided. There is no supplementary data table summarising the inputs used for meta-analysis, nor is there a public repository link or access to analysis code. This lack of raw data and transparency hinders reproducibility and limits the scientific value of the review.
The authors should include a detailed table listing study-level data extracted for the meta-analysis (e.g., sample sizes, effect sizes, standard errors), and ideally upload the dataset to a public repository (e.g., OSF, Figshare). They should also consider sharing the statistical code used for analysis to enhance transparency and reproducibility in line with PLOS ONE’s data policy.
In summary, while the manuscript now presents a clearer and more technically sound review, further improvements are needed in the justification and reporting of statistical methods and in meeting open data standards to ensure full transparency and reliability of the findings.

**Response: Thank you sincerely for your valuable and thoughtful feedback. In response to your concerns, we have supplied all the necessary supplementary materials required to support the comprehensive analysis presented in this systematic review and meta-analysis. These additional details ensure the transparency and completeness of our work, facilitating a deeper understanding and validation of our findings.**

**Review 3:**

**#1.** The authors have addressed many reviewer concerns and made commendable progress in revising the manuscript; however, several methodological, reporting, and clarity issues persist, particularly surrounding heterogeneity handling, framing of pooled estimates, language quality, and overgeneralization based on data from a few countries. The high heterogeneity remains a serious limitation to the pooled estimate’s reliability. While a meta-regression was conducted and a table provided the results, the interpretation is not sufficiently detailed in the Results or Discussion. Include a clear interpretation of key moderators. Over 60% of the included studies are from Ethiopia. While SSA is in the title and rationale, this regional label gives a false sense of geographic representativeness. Either adjust the title to reflect “Eastern Africa” or reframe the findings throughout to highlight this imbalance. Authors state “≥50% of recommended practices” as the threshold, but this criterion is inconsistent across studies. Although improvements were made, multiple instances of awkward phrasing, grammatical errors, and repetition persist.

**Response: Thank you very much for your valuable and constructive feedback, which has helped improve the quality of our manuscript. We have employed several strategies to address heterogeneity among the included studies. These include statistical tests for heterogeneity such as Cochran’s Q test and the I² statistic, and the use of an appropriate meta-analytic approach—a random effects model—due to heterogeneity levels exceeding 50%. Additionally, we explored sources of heterogeneity through subgroup analyses, meta-regression, and sensitivity analyses.**

**Regarding the framing of the pooled estimate, this systematic review and meta-analysis were conducted following standard guidelines, including clearly defining the research question, data extraction, and quality assessment protocols. Concerning language quality, we have carefully revised the entire manuscript to address language issues and improve clarity.**

**On the topic of overgeneralization, it should be noted that inclusion criteria were pre-established to minimize bias. While Sub-Saharan Africa comprises nearly 49 countries, our review includes studies from only nine countries across different regions of Africa, as determined by our protocol before the literature search. Therefore, we could not alter the study scope afterwards. Most included studies are from East Africa, particularly Ethiopia, but we also incorporated research from Western and Southern Africa. Based on this, we believe changing the title to focus solely on East Africa is not appropriate; however, we acknowledge this as a limitation in our review. The issue of high heterogeneity remains, with an I² of 96.8%, which is a significant concern in the manuscript through detailed explanation in the results and discussion sections. Regarding outcome measurement, although there were variations in measurement tools, most studies classified good practices as scores of 50% or above. Finally, we have made extensive efforts to correct phrasing, grammatical errors, and remove redundant ideas throughout the revised manuscript to enhance readability and quality.**
